# The Sphere of Institutional Treatment

**Published:** 1913-02-01

**Authors:** 


### III. THE SPHERE OF INSTITUTIONAL TREATMENT.

### Medical Attendance on a Collectivist Basis.

BY A MEDICAL SUPERINTENDENT.

One of the things which have given French prose its reputation is the frequent use of pithy interim recapitulations. Let us not be afraid of that device; for there is so much to be read nowadays that the writer who wants to hold attention must needs reiterate. In two previous articles we had got that a *Times* leader-writer, probably anxious, like most journalists, to write pleasantly, and, like every sensible person, to find something good about the all-powerful working man, said that the early acceptance of the Insurance Act by colliery doctors was due to the good system of popular control under which they worked, and they were so much in love with it. There was then duly quoted from several sources the esoterically familiar evidence to the contrary. If a little gusto appeared in doing this—in giving "bitter, old and wrinkled truth" in place of thin optimism—it was excusable considering the striking error, on the part of able people, one had come upon. We next argued that an important source of the evils disclosed was the whole set-out of the arrangements to satisfy the blind desires of the individual patient. He wants medicine; give it him then; fill up the surgery with bottles of stock mixtures, and the sick person with their contents. Again, he will not, being ignorant, miss physical examination: a good thing too, because there are over many to examine; therefore practically omit it, and pass the cases of serious illness on elsewhere. Latest of all we had got to adumbrating (Mr. Balfour is thanked for the word) our remedy, namely, to invoke in this particular case also the slow but sure tendency to transfer the business of sick attendance from the patient's home to the hospital. It falls now to consider this remedy in detail.

Remember that in reality the circumstances of the particular case are remarkably favourable: Large homogeneous communities with common employers and common interests, in every way closely knit; living densely on small neighbouring areas; accustomed, for in the North, at any rate, the co-operative movement is strong, to joint action in securing the supply of necessaries. In these democratic times, too, with great practical power of getting their own way. To call it Utopian, therefore, to picture in Lanarkshire, or Ashington, or Cannock Chase, or the Rhondda, a central medical institute for the sick population of the neighbouring coalfields, and to house temporarily most of them—to call this prospect Utopian is nonsense. A good beginning might be made with a lying-in home, to be called by some more genteel name. During each woman's stay her food ration, suitably modified to her needs, would be supplied to the home instead of to hers; and that rather dubious boon, the registered district midwife, would give way in time to a trained nurse. If we get the maternity block established we are in the position of a fresh doctor who has given satisfaction in attending a confinement. He is pretty sure to become the family attendant. We have equally earned confidence in an important quarter, for to please the collier's wife is just as important as to please the colliers' leaders. In rural localities what would be wanted next would be a fever block for the children. This will help in the same direction of getting the women's support, but its main use is securing financial resources. We shall relieve the fever hospitals proper of a few of their patients, and shall expect accordingly the support due from the rates. A special feature would be those well separated up isolation wards, with plenty of observation windows, which are now found in every big general hospital, for the accommodation of undiagnosed cases. The school clinic would find a home—as paying guest—with us too. Running ears would get a fair chance of healing under syringing, insufflation, and instillation properly done in 100 instead of about 5 per cent. of cases, with corresponding reduction in the number that have to go on to a mastoid operation. Not that the staff would boggle at tackling "mastoids," or much more serious affairs, indeed, given the safeguard of proper care and after-treatment in an institution.

### Local Model Centres.

Very soon each member would be developing his speciality or bent, gynæcological, laryngological, or whatever it might be, the cases to be picked from the large out-patient department. Of course, in ailments of sudden onset domiciliary visits would have to be paid, but the great consideration to put before patients or committeemen would be contained in this appeal:—"Come to the hospital—*your* hospital, Mr. Lachie Thompson or Mr. Geordie Robson or Mr. David Davies; believe me, in all sincerity, I can do you ever so much more good there: I can study your case, I can use special apparatus and instruments, I can guarantee, if you have to be bedfast, air space and good nursing. Your food will cost you no more there than here; you will be occupying your own house almost as much there as here; and you will save paying for my time going up and down the rows and knocking at the doors. On the road a medical man is doing no good to anybody." In the rare cases when it became necessary to exact what in the army are called "hospital stoppages," the man to be mulcted would be the man who without good reason stayed out, not the one who came in. As to building expenses, one might reckon on a good proportion of the very large contributions now paid by miners to distant general hospitals. We rather fancy such a powerful humanising influence as a local model hospital would get support, too, from mine owners, or, in the event of recently discussed measures as to nationalisation actually coming to something, from a paternal Government. Wards for higher officials might pay for themselves before long.

For an opportunity such as the above, an opportunity of a certain income, coupled with interesting work, and conditions actively fostering, not hindering, self-respect and the respect of others, would attract medical men of that class of which, to take one instance, many can be found in the nearer London suburbs; to wit, well qualified men—men not afraid of surgery, but whose capital only suffices for their education, and who therefore (for that besides they do not suffer from gentility-on-the-brain and lack supple backs) can never hope to become consultants as we now see them. Having a monopoly of the clinical material, their considered opinions on the occupational ailments of miners would be authoritative. And thus the medical attendance of the people on a collectivist basis might be worthily done. Yes, when Mr. Lloyd George set out to organise a scheme of domiciliary visiting, he was backing the wrong horse. He should, while he was about it, have made favourite the institution.

"But still, as matters are, your scheme falls foul altogether of National Insurance, let alone the Public Health Act." Is anyone so simple as to raise this objection? What do a dozen Acts of Parliament matter, nowadays, if a numerous body of working men think they could be easily modified a little to suit them better, and begin to frame demands?

